# Supplementary material for: PhosX: data-driven kinase activity inference from phosphoproteomics experiments
Source: Bioinformatics. 2024 Nov 19;40(12):btae697. doi: 10.1093/bioinformatics/btae697 (PMC11630834; doi:10.1093/bioinformatics/btae697)
Supplement: btae697_Supplementary_Data [file btae697_supplementary_data.pdf]

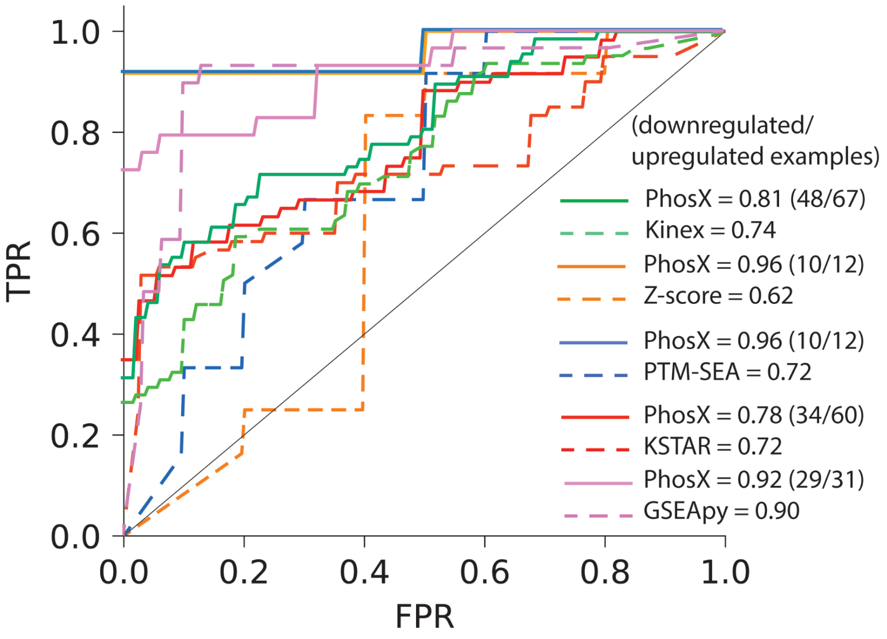

Supplementary Figure 1: Performance of the assessed methods in linearly separate upregulated from downregulated kinases in the benchmark dataset, measured by Receiver Operating curves.

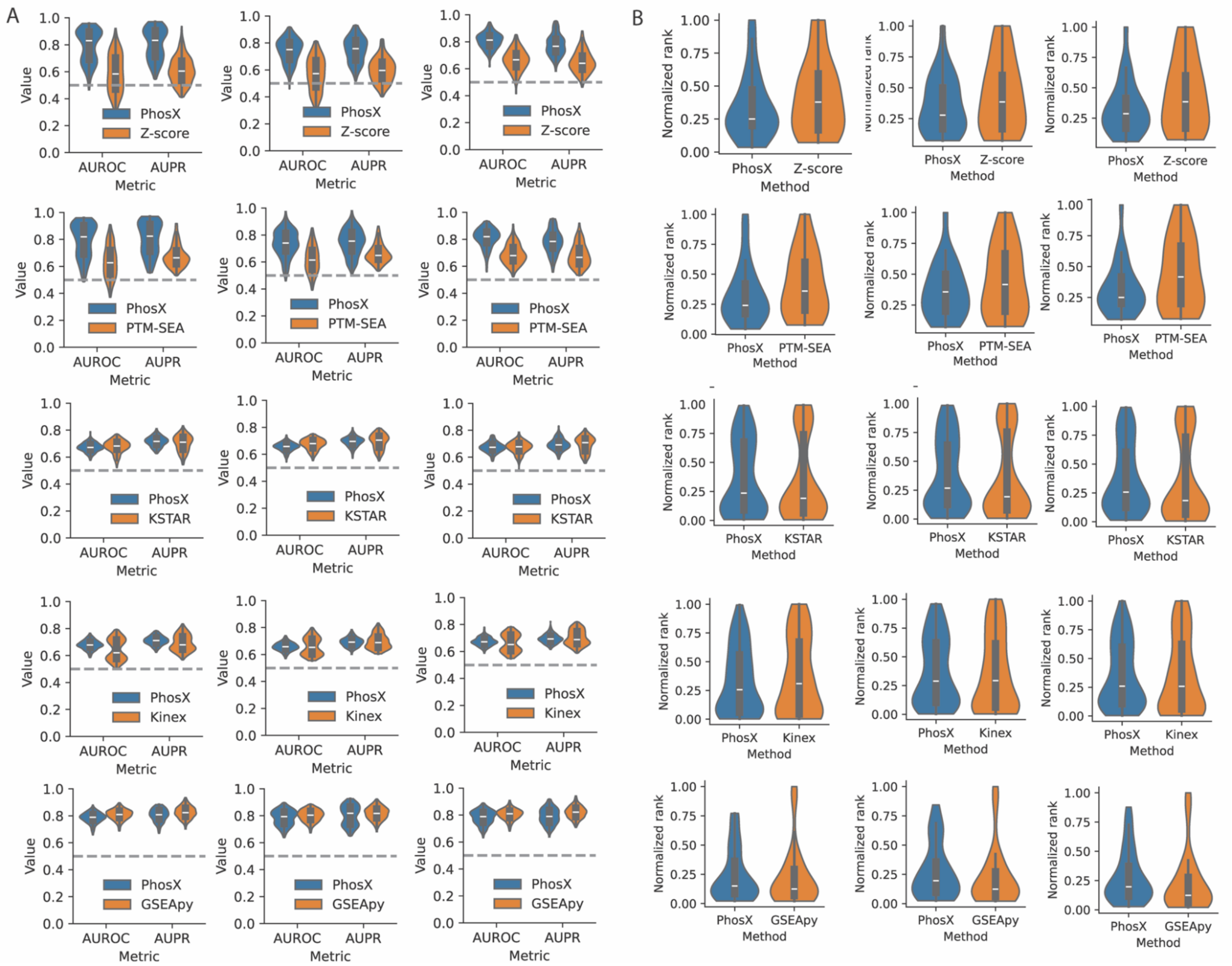

Supplementary Figure 2: Comparison of performance metrics when picking the top 5,10 and 15 kinases respectively to the columns from left to right **A.** AUROC and AUPR for separating true hits from a random negative set of kinases of equal size in the benchmark dataset. **B.** Normalised rank of the true kinases in the predictions made by PhosX and the methods it is compared to.

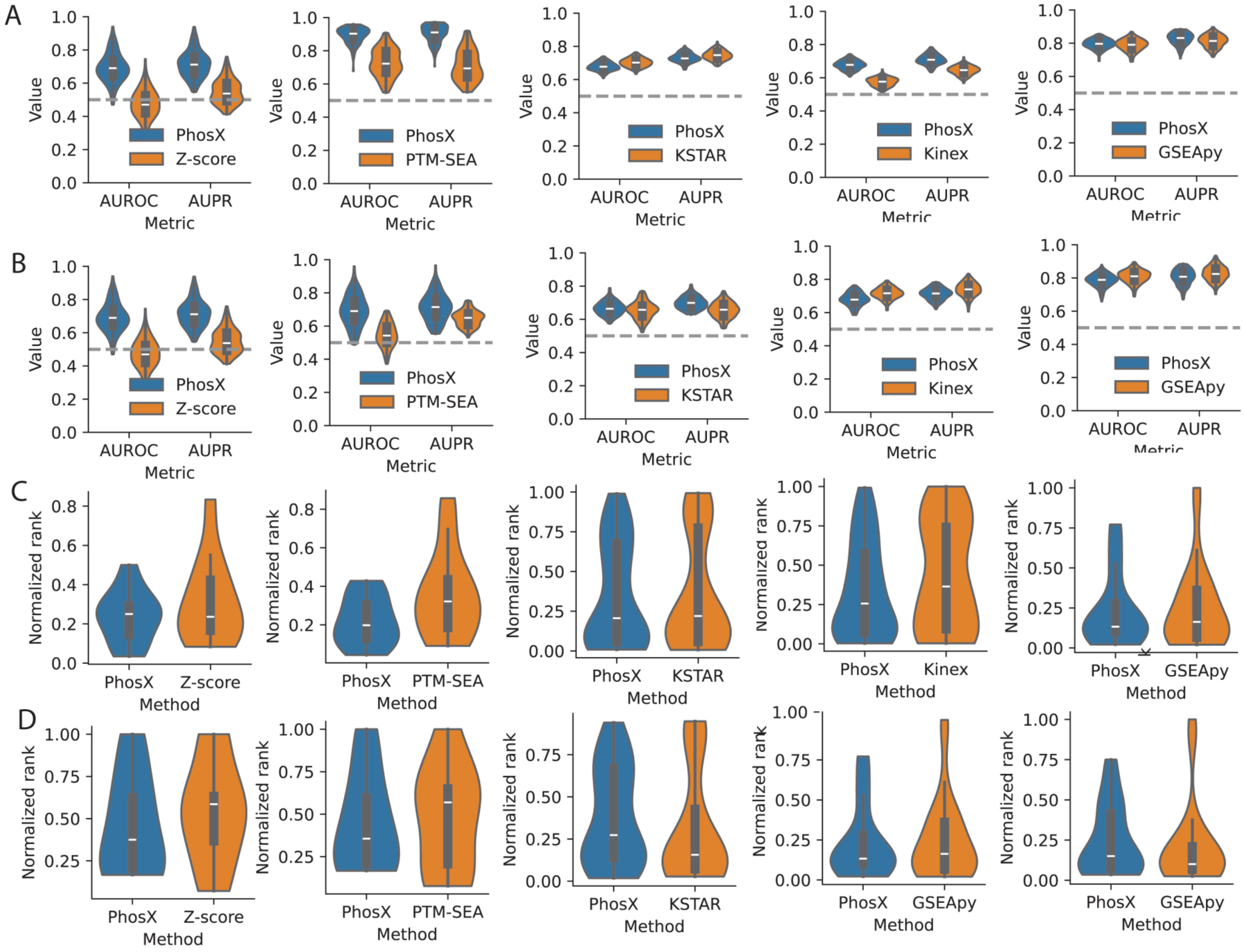

Supplementary Figure 3: **A,B.** Performance as measured by AUROC and AUPR of the assessed methods in linearly separate upregulated kinases (A) and downregulated kinases (B) from a random negative set of kinases of equal size in the benchmark dataset. **C,D.** Normalised rank of the true upregulated (C) and downregulated (D) kinases in the predictions made by PhosX and the methods it is compared to.

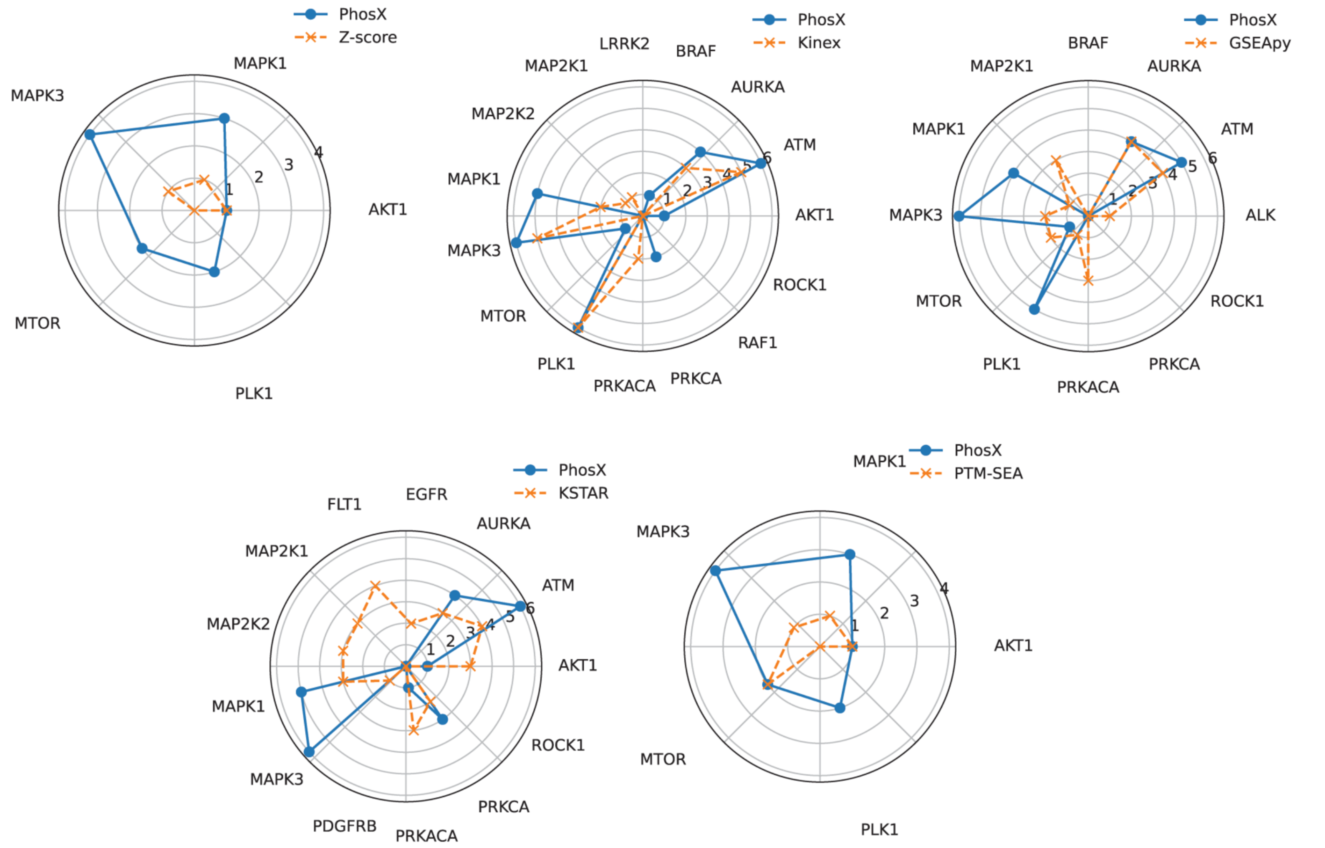

Supplementary Figure 4: Comparison for kinase-specific results between PhosX and considered methods. Number of experiments in which the specific kinase was expected to be differentially regulated and was in the extreme of the normalised activity score distribution (¿95% for upregulation and ¡5% for downregulation).

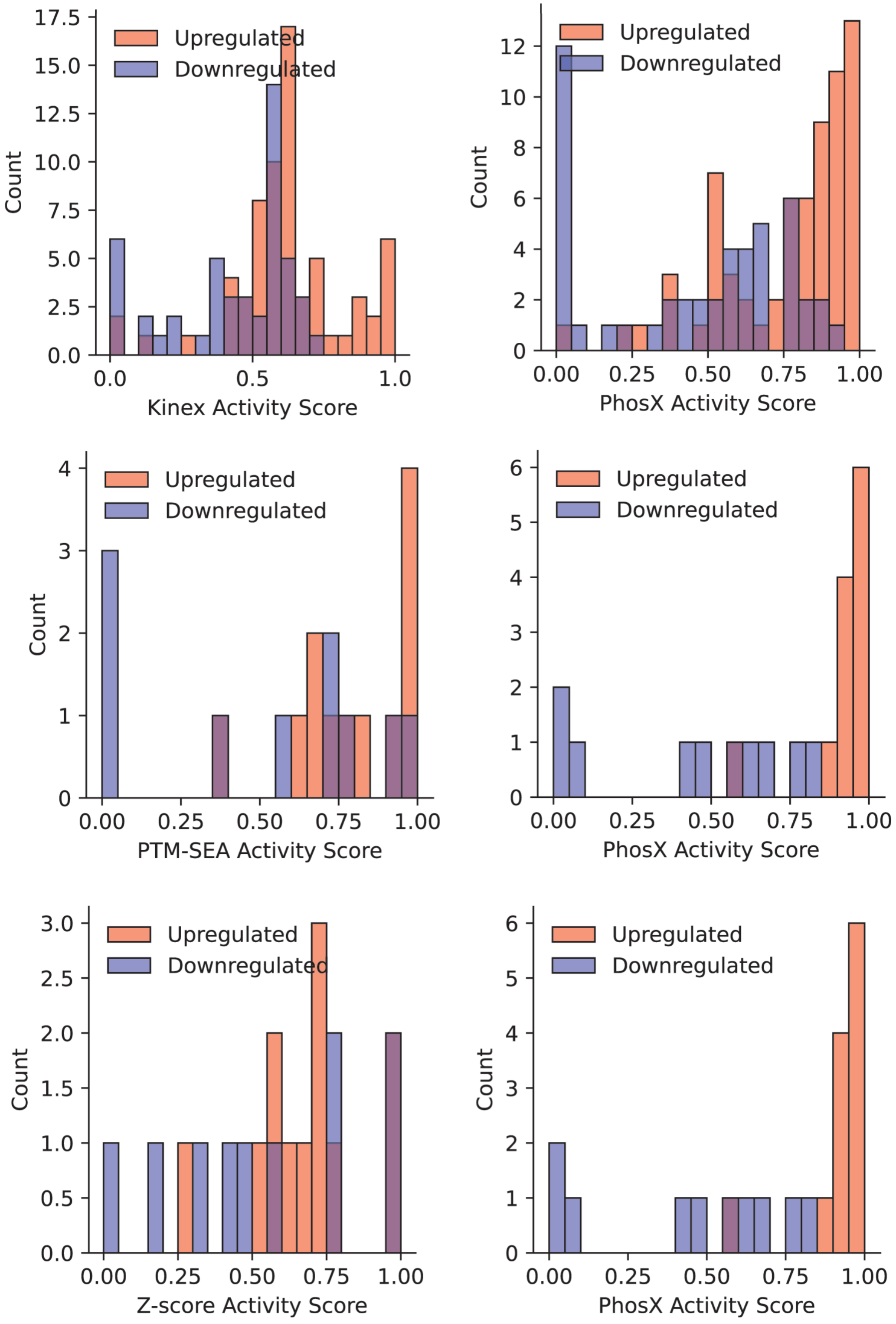

Supplementary Figure 5: Comparison of the distribution of scaled kinase activity scores of PhosX for up- and down-regulated kinases for those from Kinex, PTM-SEA and z-score.

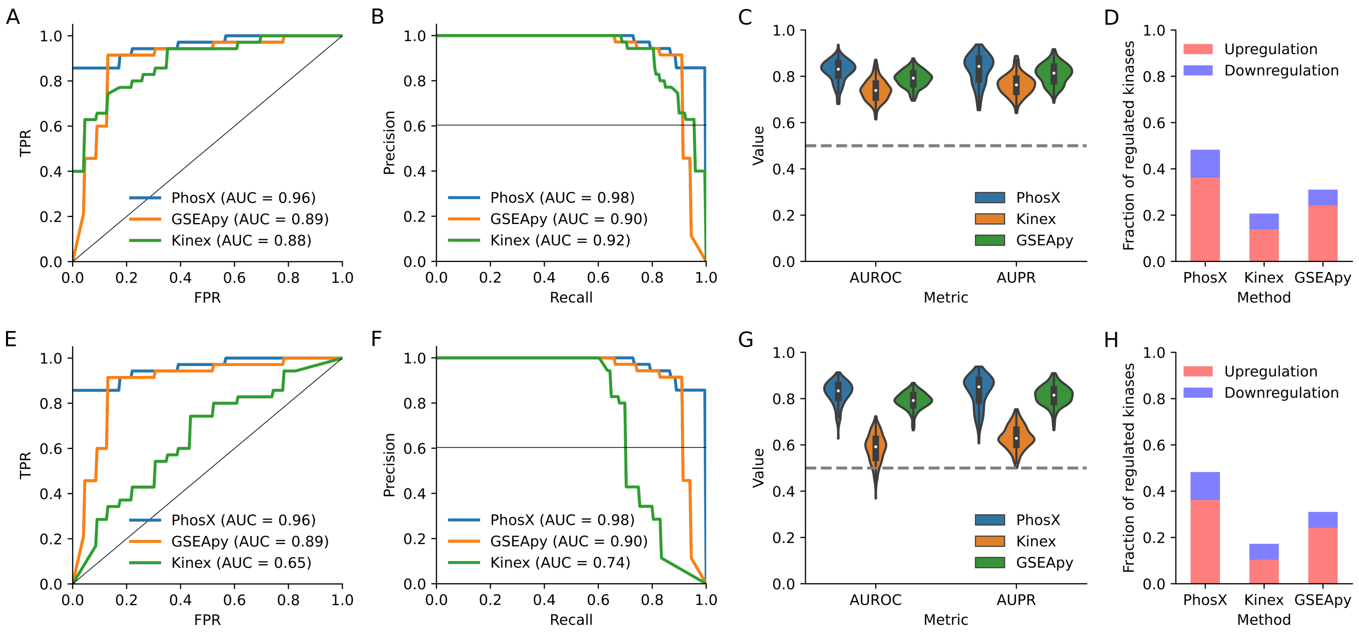

Supplementary Figure 6: Benchmark results using alternative parameter values for Kinex's log fold change (LFC) thresholds. **A.-D.** Figures refer to Kinex's LFC $= (1, -1)$. **E.-H.** Figures refer to Kinex's LFC $= (1.5, -1.5)$. **A., B., E., F.** Performance of the assessed methods in linearly separate upregulated from downregulated kinases in the benchmark dataset, measured by Receiver Operating Characteristic curves (A, E.) and Precision-Recall curves (B., F.). **C., G.** Performance of the assessed methods in linearly separate regulated kinases from a random negative set of kinases of equal size in the benchmark dataset. **D., H.** Fraction of instances of regulated kinases in the benchmark dataset found in the top-5 (upregulation) or bottom-5 (downregulation) percentiles of activity scores.
